# Pathogen seasonality and links with weather in England and Wales: a big data time series analysis

**DOI:** 10.1186/s12889-018-5931-6

**Published:** 2018-08-28

**Authors:** Mark P. C. Cherrie, Gordon Nichols, Gianni Lo Iacono, Christophe Sarran, Shakoor Hajat, Lora E. Fleming

**Affiliations:** 1 Centre for Research on Environment, Society and Health (CRESH), University of Edinburgh, Edinburgh, Scotland EH8 9XP UK; 2 Exeter University, Exeter, England; 3 Surrey University, Guildford, England; 4 Met Office, Exeter, England; 5 London School of Hygiene and Tropical Medicine, London, England; 6 European Centre for Environment and Human Health, University of Exeter Medical School, Truro, England

**Keywords:** Epidemiology, Laboratory surveillance, Statistics, Pathogen, Weather, Time-series, Salmonella

## Abstract

**Background:**

Many infectious diseases of public health importance display annual seasonal patterns in their incidence. We aimed to systematically document the seasonality of several human infectious disease pathogens in England and Wales, highlighting those organisms that appear weather-sensitive and therefore may be influenced by climate change in the future.

**Methods:**

Data on infections in England and Wales from 1989 to 2014 were extracted from the Public Health England (PHE) SGSS surveillance database. We conducted a weekly, monthly and quarterly time series analysis of 277 pathogen serotypes. Each organism’s time series was forecasted using the TBATS package in R, with seasonality detected using model fit statistics. Meteorological data hosted on the MEDMI Platform were extracted at a monthly resolution for 2001–2011. The organisms were then clustered by K-means into two groups based on cross correlation coefficients with the weather variables.

**Results:**

Examination of 12.9 million infection episodes found seasonal components in 91/277 (33%) organism serotypes. Salmonella showed seasonal and non-seasonal serotypes. These results were visualised in an online Rshiny application. Seasonal organisms were then clustered into two groups based on their correlations with weather. Group 1 had positive correlations with temperature (max, mean and min), sunshine and vapour pressure and inverse correlations with mean wind speed, relative humidity, ground frost and air frost. Group 2 had the opposite but also slight positive correlations with rainfall (mm, > 1 mm, > 10 mm).

**Conclusions:**

The detection of seasonality in pathogen time series data and the identification of relevant weather predictors can improve forecasting and public health planning. Big data analytics and online visualisation allow the relationship between pathogen incidence and weather patterns to be clarified.

**Electronic supplementary material:**

The online version of this article (10.1186/s12889-018-5931-6) contains supplementary material, which is available to authorized users.

## Background

Seasonality can be defined as increased or decreased observations that display a periodic pattern (e.g. week, month, quarter) of occurrence between years [1]. Microbial pathogens tend to be defined as microorganisms that can cause disease in humans and other organisms [2]. Reviews of their seasonality have been published previously [3]. Seasonal drivers are already known to produce annual peaks for a number of infectious diseases, including malaria [4], West Nile virus [5], and cholera [6], as well as several pathogens transmissible by contact such as influenza [7], respiratory syncytial virus [8] and Meningococcal meningitis [9].

Seasonality may be explained by a mixture of factors including climate, social, behavioural, agricultural, environmental, stochastic changes in immune populations, and other drivers. In addition, weather can influence vector abundance, pathogen survival and host characteristics (e.g. behaviour and immune susceptibility) [3]. The mathematical approaches to modelling have been reviewed [10].

Several studies have investigated the effects of weather and climate on pathogens in England and Wales. *Salmonella* Enteritidis incidence was shown to increase by 12.5% (95%CI; 11.6–13.4) for every 1 °C rise over a 6 °C threshold [11]. Similarly, Campylobacter prevalence was associated with temperature in the previous 2 weeks [12] while other studies found little association [13].

A systematic approach to the analysis of the potential seasonality of common pathogen serotypes and their associations with multiple weather variables is required to help narrow the focus on candidate pathogens in addition to those that have been studied in depth previously. The current analysis is well placed to address this gap given the rich data now available on a broad number of pathogens and meteorological factors. The aim of the analysis was to use several data mining techniques to identify pathogens that display a seasonal component, and describe their associations with meteorological factors as an aid to future analytical work (including forecasting) and public health planning.

## Methods

### Infectious disease data

Infectious disease data from England and Wales were collected by Public Health England (PHE) (formerly the Health Protection Agency and before that the Public Health Laboratory Service) through a voluntary reporting system, whereby hospital laboratory records are transferred to regional epidemiology units, processed and added to the LabBase2 national surveillance database [14]. To avoid duplication by organism and patient, each record has a unique identifier called the Organism Patient Illness Record (OPIE). If a record is sent with the same patient and organism information within 14 days (26 weeks for *Mycobacterium* spp.), then these cases are merged to ensure a single OPIE for the entire duration of the episode. The Second Generation Surveillance System (SGSS- formerly LabBase2) voluntary national surveillance database holds records on 12,904,446 reportable human infectious cases spanning from the 1st week in 1988 to the 2nd week in 2015 for 344 root organisms and 2014 serotypes. Pathogen counts were recorded at a weekly level in the database. The analysis for individual serotypes was restricted to complete years, from 1989 to 2014, with serotypes greater than 854 cases (above quartile one, i.e. top 25% in terms of total count), as a time series model could not be automatically estimated with fewer cases (*n* = 277). We aggregated the data to a monthly level and linked with national meteorological data held on the Medical and Environmental Data Mash-up Infrastructure project (MEDMI) platform [15]. The analysis was performed at a national scale due to multiple factors at a local level that act as noise to obfuscate the relationship between infectious disease and weather [16].

### Meteorological data

A range of meteorological data for the UK was downloaded from the MEDMI Platform [15] at a 5 km by 5 km resolution for 2001–2011; full details on methods used to generate data are provided elsewhere [17]. The variables were monthly weather summaries that included: mean sunshine duration (hours per day), mean temperature (°C), mean daily maximum temperature (°C), mean daily minimum temperature (°C), mean vapour pressure (hPa), mean sea level (MSL) pressure (hPa), rain ≥1 mm (days), rain ≥10 mm (days), total rainfall (mm), mean wind speed at a height of 10 m (knots), mean relative humidity (%), snow lying over 50% of ground (days), ground frost measured as grass minimum temperature below 0 °C (days), and air frost measured as air minimum temperature below 0 °C (days) (Additional file 1: Figure S1). The data were imported into ArcMap 10 (ESRI, Redwoods, CA) and aggregated (arithmetic mean) for England and Wales, which enabled linkage with the infectious disease time series data.

### Statistical analyses

Descriptive statistics were generated for the organisms including total count, crude prevalence rate per month, peak month and plots of time-series patterns (for gastro-intestinally acquired infections and those from respiratory transmission). We applied a two stage automated analysis to: a) detect seasonality and b) identify correlations with weather variables. The first stage was the seasonality detection analysis, undertaken in Rstudio (ver 0.98.507). Description of the forecast package, which was used extensively in the analysis to automatically detect seasonal patterns, has been detailed elsewhere [18]. Briefly, the pathogen time series data were decomposed via Box Cox Transformations into trend, seasonal and irregular components, which were used to forecast the time series into the future [18]. The algorithm automatically selects model parameters such as trend (with or without a dampening parameter) and noise (ARMA (p,q) process) using model fit statistics (i.e. minimising Akaike Information Criteria (AIC)). A TBATS model, as described above, was fitted for each organism serotype (with a non-zero count) using the weekly periodicity (i.e. the most granular temporal resolution available). The models were re-run with data aggregated at monthly and quarterly periodicities to investigate seasonality at different temporal aggregations [19]. Each time the model would provide a logical output (i.e. true/false) as to whether the model fit improved with the inclusion of the seasonal component (i.e. consistent repeating pattern over time). This is because the algorithm fits two models, seasonal and non-seasonal, and selects the seasonal model if the AIC is lower than the non-seasonal model (heuristically, it selects the model that results in the best combination of good fit and lower number of parameters). To limit the seasonality definition to those whose model fit was significantly better with the addition of the seasonal component, we calculated the difference between the seasonal and non-seasonal AIC (*∆*_*i*_ = *AIC*_*nonseasonal*_ − *AIC*_*seasonal*_) and excluded organisms with AIC difference greater than 10, as suggested as a suitable cut-off by Burnham and Anderson [20]. The pathogens at a monthly resolution with AIC difference greater than 10 were used in subsequent analysis with weather variables.

For the second stage, we aggregated the pathogen incidence data to monthly resolution so that they were able to be merged with the weather variables previously processed to monthly values by the National Climate Information Centre. The time series’ for each of the weather variables was shown to be stationary (no significant trend from year to year) by using the Augmented Dickey–Fuller (AF) test (*p* < 0.05) and Kwiatkowski-Phillips-Schmidt-Shin (KPSS) test (*p* > 0.05). We tested each pathogen time series in the same way. Some were found to be non-stationary and differenced (once or twice, depending on results of AF and KPSS tests). Cross correlation coefficients were generated between cases and weather variables for the month that they were recorded and then by the meteorological values lagged by 1 month. The correlation coefficients were then used as input to the K-means clustering method. Two clusters were generated in order to narrow the focus on those correlated with weather. The terminology for discussing the correlation coefficients was as follows: very weak (*r* = 0–0.19), weak (*r* = 0.20–0.39), moderate (*r* = 0.40–0.59), strong (0.60–0.79) and very strong (*r* = 0.80–1.00). Seasonality and weather correlation results were summarised and discussed in terms of differences between weather variables and within the most common genus for which serotypes were available (*Salmonella*).

### Data visualisation

Supplementary to the time series analysis, an Rshiny app was developed to display the results and aid future hypothesis generation. The user can filter the pathogens by seasonality, prevalence and serotype. Once an individual serotype is selected, a range of descriptive information is available: Wikipedia description, total number of cases, time series plot, month plot of crude rate per 100,000 (England and Wales population), decomposition of time series, TBATS model forecast and weather scatterplot.

## Results

### Descriptive results- pathogens

The weekly data on 12.9 million pathogen infections in England and Wales from 1989 to 2014 were examined systematically. The minimum number for an organism to be in the database during the time period was once per week. The maximum number of cases for 1 week was 4073 for *Chlamydia trachomatis*. There was a non-normal distribution of total cases, from one case for 345 organisms to 2,094,656 for *Chlamydia trachomatis*. The median number of total cases was 3156 (Interquartile range quartile 1- quartile 3; 854–15,730). The organisms with the highest number of serotypes were Salmonella (*n* = 890) and Streptococcus (*n* = 60), although most of these had very low counts.

**Fig. 1:**
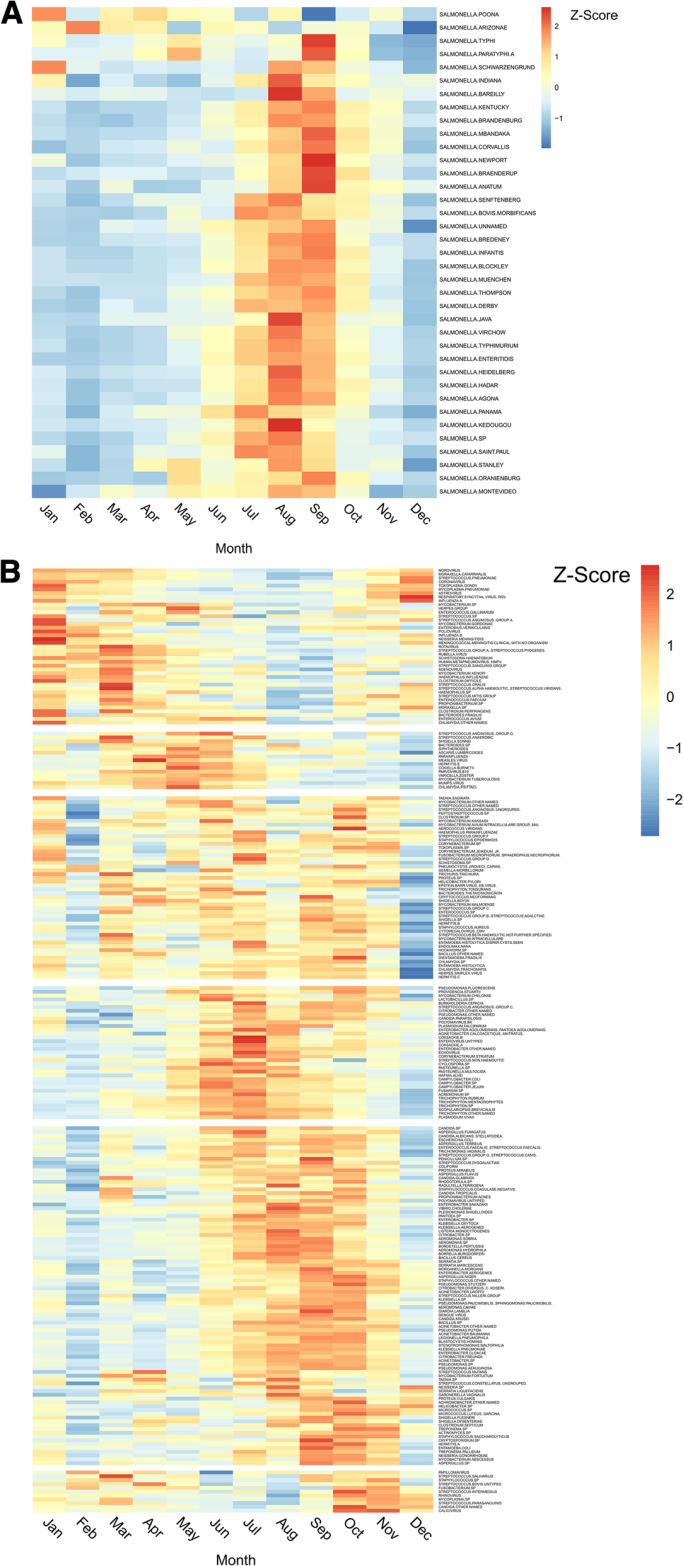
**a**: Distribution of z-score salmonella pathogen crude rates by month. **b**: Distribution of z-score non-salmonella pathogen crude rates by month

**Fig. 2:**
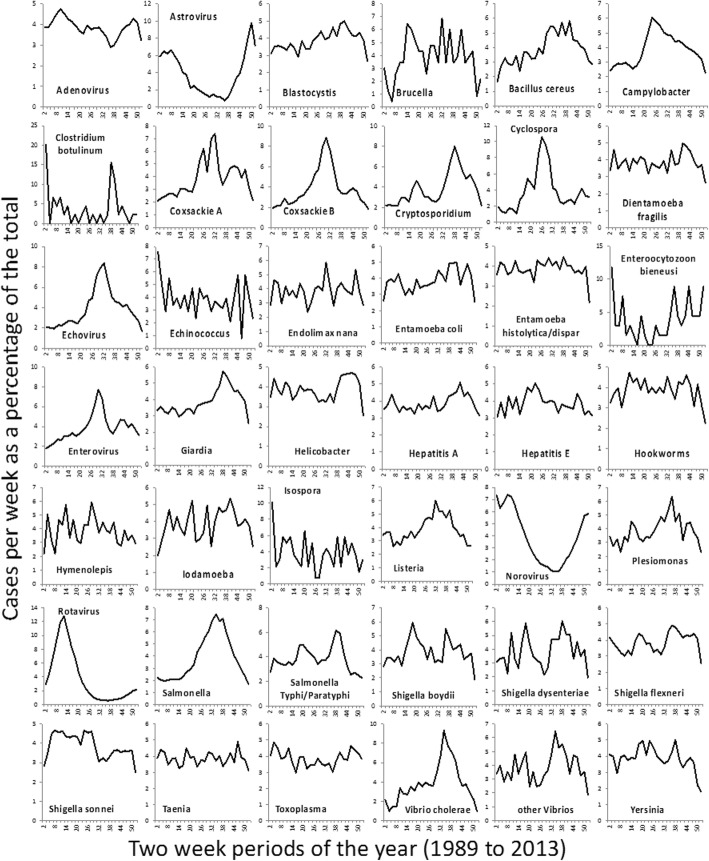
Seasonal distribution of gastro-intestinally transmitted pathogens

**Fig. 3:**
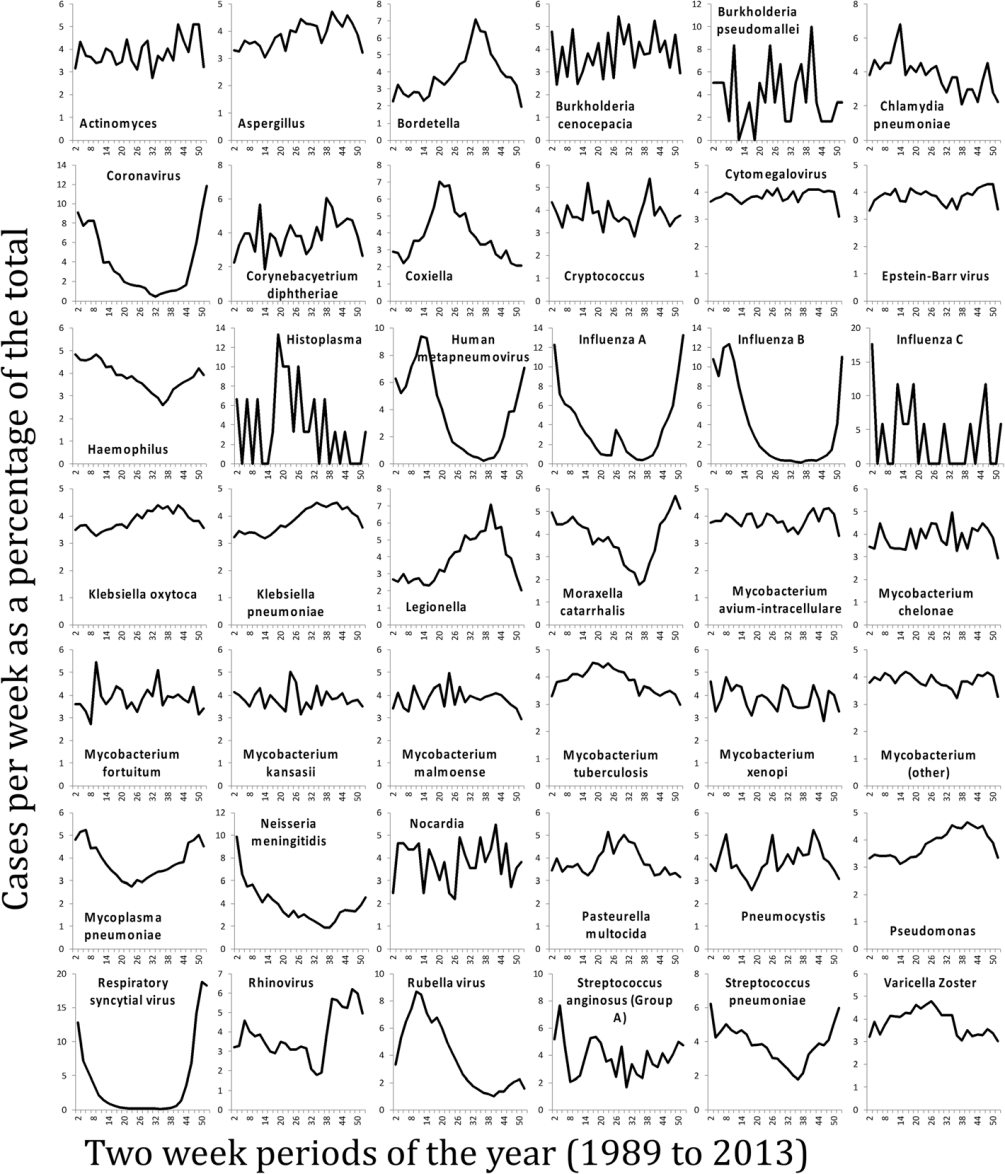
Seasonal distribution of respiratory tract transmitted pathogens

Figure 1 shows a heat map of z-scores of crude rates by month (Fig. 1 shows non-*salmonella* pathogens, and Fig. 1 shows only the *Salmonella* genus). The months with the fewest high pathogen rates for the majority of organisms were December (36.1%) and February (31.4%). The months with the highest number of high pathogen rates were more evenly spread out over the summer and autumn, with July, August, September and October being the highest months for 62.2% of the organisms. The seasonality of gastro-intestinally acquired infections (Fig. 2), and pathogens acquired through respiratory transmission (Fig. 3), differed substantially. The gastro-intestinal pathogens showed different distributions, with most bacteria having higher rates in summer, some viruses had higher rates in winter (e.g. norovirus, rotavirus) and others were more common in the summer (enteroviruses). Some of the pathogens associated with travel overseas had a late summer increase (thought to reflect the period when people return from summer holidays). The respiratory pathogens predominated in the winter months (e.g. coronavirus, influenza, Respiratory Syncytial Virus (RSV)). However, several of the bacterial pathogens were more frequent in warmer months (e.g. *Bordetella*, *Coxiella*, *Legionella*).

### Seasonality detection and association with weather variables

**Fig. 4:**
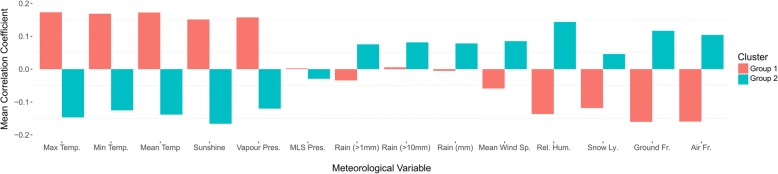
K-means clusters of pathogens by correlation with meteorological variables

We detected significant seasonality in 91 organisms using TBATS models at varying periodicities (91/277; 33%) (Additional file 2: Table S1); with varying links with weather (Additional file 3: Figure S2). Two k-means clusters (identified as the optimum number of k) were generated from the cross correlation coefficients with weather variables and represented groups of pathogens that had similar correlations with weather variables (Fig. 4). The two groups were characterised by their relationship with the weather variables (Additional file 4: Table S2). Group 1 had mean positive correlations with higher temperature (min, mean, max), sunshine and vapour pressure; whilst the Group 2 had positive mean correlations with lower temperature variables (snow lying, ground frost, air frost), precipitation (rain days over 1 mm, rain days over 10 mm and rainfall), mean wind speed and relative humidity. Within Group 1 there were pathogens with the strongest correlations with sunshine (*n* = 25) and vapour pressure (*n* = 11). In Group 2, pathogens had highest correlations with relative humidity (*n* = 8) and Ground frost (*n* = 5) (Additional file 5: Figure S3). There was at least one pathogen with the highest correlation for each meteorological variable. Summary information on seasonality and links with weather, by temperature cluster group are presented in Table 1.

**Table 1:** Summary table of seasonal pathogens with potential links with weather

Genus	Serotype	Weather variable max correlation name (lag 1 month)	Weather variable max correlation (lag 1 month)	Weather Group	Weather variable min correlation name (lag 1 month)	Weather variable min correlation (lag 1 month)
ACINETOBACTER	BAUMANNII	Sunshine	0.23	1	Relative.humidity	−0.01
ACINETOBACTER	SP	Min.temp	0.2	1	Ground.frost	−0.09
ACINETOBACTER	LWOFFII	Mean.temp	0.13	1	Ground.frost	−0.06
ADENOVIRUS		Raindays.10	0.15	2	Sunshine	−0.06
AEROMONAS	HYDROPHILA	Sunshine	0.32	1	Relative.humidity	−0.15
AEROMONAS	SP	Sunshine	0.28	1	Relative.humidity	−0.03
AEROMONAS	SOBRIA	Max.temp	0.18	1	Rainfall	−0.09
ASPERGILLUS	FLAVUS	Min.temp	0.13	1	Air.frost	−0.01
ASTROVIRUS		Relative.humidity	0.2	2	Sunshine	−0.01
BACILLUS	CEREUS	Sunshine	0.25	1	Relative.humidity	−0.08
BACILLUS	SP	Sunshine	0.17	1	Relative.humidity	−0.05
BLASTOCYSTIS	HOMINIS	Mean.temp	0.19	1	Ground.frost	0
BORDETELLA	PERTUSSIS	Sunshine	0.23	1	Relative.humidity	− 0.02
BORRELIA	BURGDORFERI	Sunshine	0.4	1	Relative.humidity	−0.04
CAMPYLOBACTER	SP	Sunshine	0.37	1	Relative.humidity	− 0.08
CAMPYLOBACTER	JEJUNI	Sunshine	0.21	1	Relative.humidity	−0.02
CANDIDA	PARAPSILOSIS	Raindays.10	0.15	1	Snow.lying	−0.09
CHLAMYDIA	SP	Raindays.1	0.11	1	Air.frost	0
CITROBACTER	SP	Vapour.pressure	0.23	1	Mean.wind.speed	−0.02
CITROBACTER	FREUNDII	Mean.temp	0.2	1	Air.frost	−0.01
COXSACKIE	B	Rainfall	0.12	1	MLSPressure	−0.06
CRYPTOSPORIDIUM	SP	Relative.humidity	0.29	2	Sunshine	0
CYCLOSPORA	SP	Sunshine	0.14	2	Relative.humidity	−0.02
ECHOVIRUS		Sunshine	0.12	1	Relative.humidity	0
ENTAMOEBA	HISTOLYTICA	Ground.frost	0.06	2	Mean.wind.speed	−0.02
ENTEROBACTER	CLOACAE	Min.temp	0.26	1	Air.frost	−0.06
ENTEROBACTER	OTHER NAMED	Max.temp	0.18	2	Snow.lying	0
ENTEROBACTER	SP	Min.temp	0.15	1	Ground.frost	−0.05
ENTEROBACTER	AGGLOMERANS (PANTOEA AGGLOMERANS)	Snow.lying	0.09	1	Relative.humidity	0
ENTEROBIUS	VERMICULARIS	Vapour.pressure	0.14	1	Ground.frost	−0.03
ENTEROVIRUS	UNTYPED	Sunshine	0.1	1	Snow.lying	−0.04
GIARDIA	LAMBLIA	Relative.humidity	0.37	2	Sunshine	−0.02
HAEMOPHILUS	INFLUENZAE	Relative.humidity	0.23	2	Sunshine	−0.01
HEPATITIS	A	Min.temp	0.2	1	Air.frost	−0.11
HEPATITIS	E	Mean.wind.speed	0.17	2	Vapour.pressure	−0.02
HERPES SIMPLEX VIRUS		Mean.wind.speed	0.11	1	Snow.lying	−0.01
HUMAN METAPNEUMOVIRUS (HMPV)		Relative.humidity	0.55	2	Sunshine	−0.09
INFLUENZA	A	Snow.lying	0.32	2	Raindays.1	−0.02
INFLUENZA	B	Ground.frost	0.21	2	Max.temp	0
LISTERIA	MONOCYTOGENES	Vapour.pressure	0.36	1	Ground.frost	−0.02
MORAXELLA	CATARRHALIS	Relative.humidity	0.26	2	Sunshine	−0.12
MYCOBACTERIUM	TUBERCULOSIS	Mean.wind.speed	0.14	2	Vapour.pressure	−0.01
MYCOPLASMA	PNEUMONIAE	Ground.frost	0.43	2	Mean.temp	−0.35
NEISSERIA	MENINGITIDIS	Relative.humidity	0.33	2	Sunshine	−0.01
PANTOEA	SP	Sunshine	0.12	1	MLSPressure	−0.03
PARAINFLUENZA		Ground.frost	0.23	2	Vapour.pressure	−0.01
PARVOVIRUS	B19	Sunshine	0.54	1	Relative.humidity	−0.11
PASTEURELLA	MULTOCIDA	Sunshine	0.09	1	Air.frost	0
PLASMODIUM	FALCIPARUM	Max.temp	0.16	1	Ground.frost	−0.01
PLESIOMONAS	SHIGELLOIDES	Vapour.pressure	0.31	1	Snow.lying	−0.01
PLESIOMONAS	AERUGINOSA	Min.temp	0.2	1	Ground.frost	−0.04
PROTEUS	VULGARIS	Vapour.pressure	0.14	1	Snow.lying	0
PSEUDOMONAS	PUTIDA	Max.temp	0.15	1	Mean.wind.speed	0
PSEUDOMONAS	OTHER NAMED	Sunshine	0.2	1	Air.frost	0
RESPIRATORY SYNCYTIAL VIRUS (RSV)		Air.frost	0.69	2	Max.temp	−0.6
RHINOVIRUS		Raindays.10	0.17	1	Snow.lying	−0.07
ROTAVIRUS		Sunshine	0.4	1	Relative.humidity	−0.03
RUBELLA VIRUS		Snow.lying	0.53	2	Mean.wind.speed	−0.03
SALMONELLA	ENTERITIDIS	Sunshine	0.52	1	Relative.humidity	−0.03
SALMONELLA	TYPHIMURIUM	Vapour.pressure	0.46	1	Relative.humidity	−0.02
SALMONELLA	KENTUCKY	Max.temp	0.37	1	Relative.humidity	−0.09
SALMONELLA	DERBY	Vapour.pressure	0.36	1	Relative.humidity	−0.12
SALMONELLA	AGONA	Vapour.pressure	0.35	1	Relative.humidity	−0.1
SALMONELLA	PARATYPHI A	Mean.temp	0.33	1	Relative.humidity	0
SALMONELLA	STANLEY	Mean.temp	0.32	1	Relative.humidity	−0.04
SALMONELLA	VIRCHOW	Sunshine	0.29	1	Relative.humidity	−0.01
SALMONELLA	THOMPSON	Raindays.10	0.28	1	Ground.frost	−0.01
SALMONELLA	INFANTIS	Relative.humidity	0.25	1	Ground.frost	−0.1
SALMONELLA	BLOCKLEY	Sunshine	0.24	1	Air.frost	−0.03
SALMONELLA	MONTEVIDEO	Sunshine	0.23	1	Air.frost	−0.02
SALMONELLA	HADAR	Sunshine	0.22	1	Mean.wind.speed	−0.06
SALMONELLA	NEWPORT	Raindays.10	0.21	2	Relative.humidity	−0.06
SALMONELLA	MBANDAKA	Vapour.pressure	0.2	1	Ground.frost	−0.06
SALMONELLA	BRANDENBURG	Vapour.pressure	0.17	1	Ground.frost	−0.09
SALMONELLA	HEIDELBERG	Sunshine	0.15	1	Ground.frost	−0.03
SALMONELLA	ORANIENBURG	Mean.wind.speed	0.14	1	MLSPressure	−0.03
SALMONELLA	JAVA	Raindays.10	0.14	1	Ground.frost	−0.02
SALMONELLA	UNNAMED	Sunshine	0.13	1	Ground.frost	−0.14
SALMONELLA	BRAENDERUP	Vapour.pressure	0.13	1	Mean.wind.speed	−0.01
SALMONELLA	TYPHI	Mean.wind.speed	0.1	1	Ground.frost	−0.11
SALMONELLA	SAINT-PAUL	Sunshine	0.08	1	Air.frost	−0.01
SERRATIA	MARCESCENS	Min.temp	0.19	1	Snow.lying	−0.04
SERRATIA	SP	Min.temp	0.17	1	Air.frost	−0.06
SHIGELLA	SONNEI	Sunshine	0.26	1	Snow.lying	−0.07
SHIGELLA	FLEXNERI	Raindays.1	0.21	2	Relative.humidity	−0.05
STENOTROPHOMONAS	MALTOPHILIA	Min.temp	0.3	1	Air.frost	0
STREPTOCOCCUS	PNEUMONIAE	Relative.humidity	0.28	2	Sunshine	−0.15
TRICHOPHYTON	SP	MLSPressure	0.17	1	Max.temp	−0.01
TRICHOPHYTON	OTHER NAMED	Mean.wind.speed	0.17	2	Relative.humidity	−0.03
TRICHOPHYTON	TONSURANS	Ground.frost	0.11	2	Max.temp	0
VARICELLA ZOSTER		Mean.wind.speed	0.13	2	Vapour.pressure	−0.03

### Pathogen weather groups

Group 1 consisted of 66 organisms, of which 22 were from the *Salmonella* genus. Parvovirus B19 had a moderate correlation with sunshine (mean *r* = 0.54), followed by *Salmonella* Enteritidis with sunshine (*r* = 0.52) and *Salmonella* Typhimurium with vapour pressure (*r* = 0.46). Group 2 consisted of 25 pathogens of which only two genus (Influenza and trychophyton) had more than one serotype. RSV had strong correlations with air frost (*r* = 0.69), followed by moderate correlations between Human metapneumovirus (HMPV) with relative humidity (*r* = 0.55) and Rubella virus with lying snow (*r* = 0.53).

### Differences between weather variables

We were interested in how the correlation coefficients varied between the weather variables that measured the same phenomenon (e.g. min, max, mean temperature). In general, there were slight differences between the different measures of temperature. The mean difference in correlation coefficients between minimum and maximum temperature was 0.002 with standard deviation of 0.02. HMPV and *Rotavirus* showed the largest difference between the temperature variables (comparing min temp and max temp). HMPV recorded a 0.14 higher coefficient for maximum temperature, whereas *Rotavirus* recorded a 0.16 higher coefficient for minimum temperature. Similar associations with temperature were found with vapour pressure and sunshine, although they tended to be relatively weaker when taking the mean for all of the pathogens There were also similar moderate inverse correlations with ground frost, air frost and snow lying days. For *Influenza A*, days with lying snow had a higher correlation than the other weather variables (*r* = 0.32). Notable differences in correlations between pathogens and the precipitation variables (comparing days with over 10 mm of rain compared to days with over 1 mm of rain), included *Plesiomonas shigelloides* with a 0.19 higher correlation with days over 10 mm and RSV with a 0.15 higher correlation with days over 1 mm of rain.

### Differences within the Salmonella genus

**Fig. 5:**
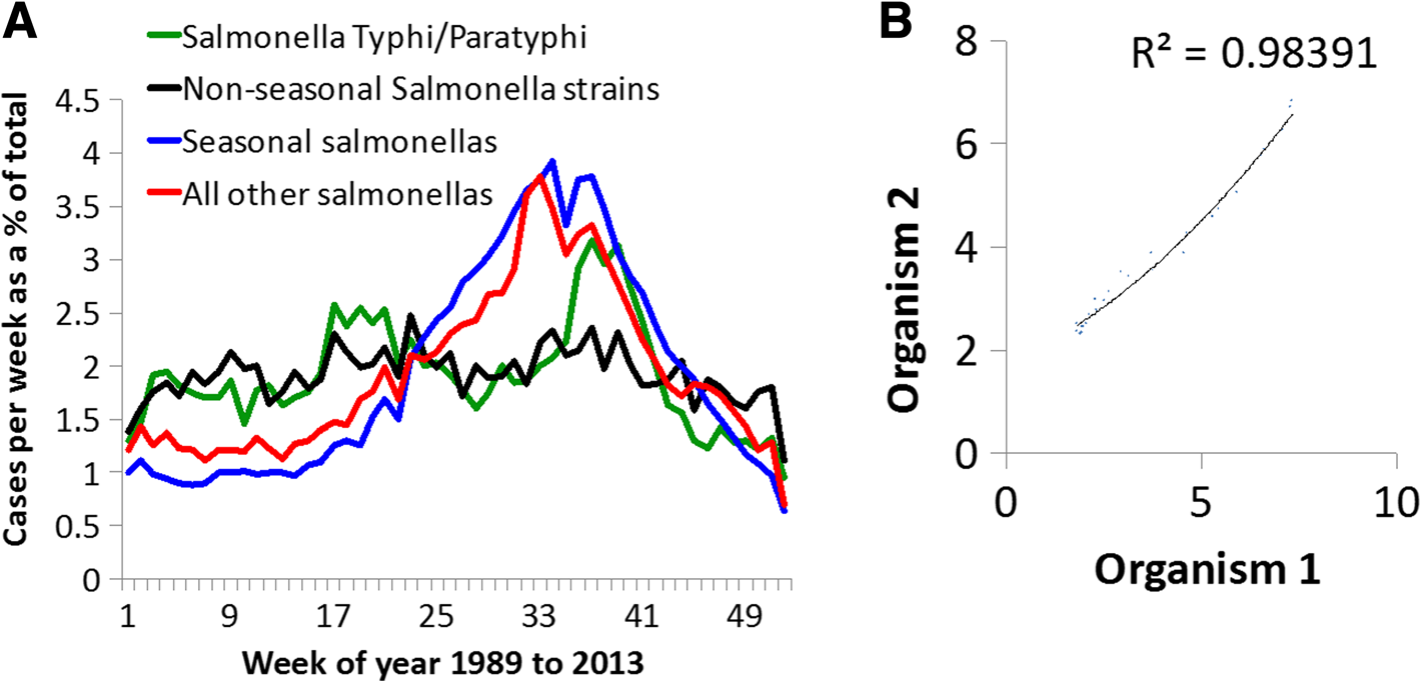
Salmonella pathogens. **a**: Comparison of 1. S. Typhi/S. Paratyphi; 2. Seasonal salmonellas; 3. Strains showing no evidence of any seasonality and 4. The remaining *Salmonella* serotypes; Seasonal serotypes: Agama, Agona, Anatum, Blockley, Bovis-Morbificans, Braenderup, Bredeney, Cerro, Coeln, Corvalis, Derby, Drypool, Duisburg, Durham, Emek, Enteritidis, Gold Coast, Grumpensis, Hadar, Haifa, Heidelberg, Ibadan, Infantis, Java, Kentuckey, Kottbus, Livingstone, London, Manchester, Manhattan, Mbandaka, Muenchen, Muenster, Napoli, Newport, Ohio, Oranienburg, Othmarschen, Panama, Saint-Paul, San-Diego, Senftenberg, Sofia, Stourbridge, Thompson, Typhimurium, Zanzibar; Non-seasonal serotypes: Adelaid, Albany, Arechavaleta, Arizonae, Colindale, Dublin, Durban, Ealing, Havana, Javiana, Marina, Mississippi, Monschaui, Montevideo, Nima, Oslo, Pomona, Poona, Rubislaw, Weltevreden, Worthington; **b**: Correlation between seasonal (group 2) and non-seasonal (group 4) salmonella pathogens

Salmonella serotypes featured heavily with varying strength and pattern of seasonality detected. *Salmonella* Enteritidis and *Salmonella* Typhimurium had the strongest associations with meteorological variables. The remaining Salmonella serotypes were split between being weakly correlated (*n* = 15) and very weakly correlated (*n* = 8). There is some reason to believe that the epidemiological causes of seasonality in most Salmonellas is similar (24/25; 96% belong to Group 1) and the association with temperature might be linked to growth in prepared foods. In addition, the strength of association in linking the seasonality or temperature to cases will be limited to the number of isolates in each serogroup. Because of this the salmonellas were grouped into four groups (1. Salmonellas causing enteric fever that are usually acquired overseas (*S.* Typhi*/S.* Paratyphi); 2. Seasonal salmonellas; 3. Strains showing no evidence of any seasonality and 4. The remaining strains where there are insufficient numbers to determine seasonality). The remaining strains included serotypes that had so few isolates that seasonality could not be determined. When grouped thus, the seasonality of the seasonal salmonellas (2) resembled that of the remaining strains (4), while the overall seasonality of serotypes that individually showed little evidence of seasonality were not obviously seasonal when combined (Fig. 5). The seasonality of groups 2 and 4 showed a high degree of correlation using data averaged over the 25-year period (r^2^ = 0.98; Fig. 5b).

## Discussion

### Principal findings

We have systematically examined a large number of human infectious disease pathogens for seasonality, and detailed potential links with weather in England and Wales. This was made possible by utilising time series and clustering algorithms that can detect patterns in the data without supervision. This can lead to greater research efficiency by defining a focus for further investigations. We found that 91 of the most prevalent organisms displayed seasonality, classified into two groups due to their association with 1 month lagged meteorological variables. Within these groups, there were well-known seasonal pathogens such as RSV, *Campylobacter* and *Salmonella*, as well as other less studied organisms such as *Aeromonas.*

### Strengths and limitations

The limitations of the big-data approach in this analysis meant that it was not possible to undertake analysis on causative weather factors on pathogen incidence. Behavioural determinants that correlate with season and weather may explain the correlations found. For example, school closures for holidays can reduce transmission and therefore cases of influenza [21], outdoor eating, when the temperature is higher increases risk of *Salmonella*, undercooking, raw meat contamination and recreational activities on water, are more likely to occur in summer, are associated with Campylobacter [22]. In separate work we are looking at methods to separate out the weather parameters from seasonality (and the associated behavioural determinants) using local weather data linkage, as described in ‘recommendations for future research’ [23]. The study was limited by the temporal and spatial aggregation of the data, and therefore we were unable to investigate the effect of day-to-day weather in regions of England and Wales. The results of the analysis were also dependent on the time-period used. For example, *C. difficile* have been reported to have a strong seasonal pattern previously using hospital episode statistics from England from 1995 to 2006 [24]; however we did not find a strong seasonal component in our study period. In our analyses, *C. difficile* displayed a peak in 2006 and then reduced in prevalence and seasonality. Therefore, the results are presented with a caveat that the correlation coefficients with weather were sensitive to the time-period under analysis and would be expected to differ in a pathogen-dependent manner.

The surveillance methods for collecting data changed over the years, with many pathogens having separate expert surveillance datasets that are independent of this data and some periods of enhanced surveillance or poor surveillance. There have also been periods where an intervention (e.g. vaccination) had been introduced, as well as those where the surveillance had improved (e.g. fungal infections; hospital infections), although we were unable to systematically account for these changes in the current analysis. Furthermore, the data were lab-confirmed and therefore do not represent milder unreported or undiagnosed cases which may display a different pattern of seasonality. Finally, we could not ascertain concomitant pathogens as they were not readily extractable from the database. The analysis was limited as it only considered a 1 month lag effect and did not consider time-varying confounders. Lag effects can vary for different environmental exposures. For example sunshine will induce 25-hydroxy-vitamin D production (the major circulating form of vitamin D) in human skin; 25-hydroxy-vitamin D will lag sunshine exposure by up to 2 months due to metabolism within the body [25]. Also, the life-cycle of the pathogen or vector varies between organisms producing a lag between weather exposure and clinical manifestations of pathogen and subsequent laboratory diagnosis [26], but this has not been addressed in the current study. Lag effects may be more pronounced for organisms that are indirectly rather than directly associated with weather [27], for example weather conditions that precede mosquito larvae growth do not immediately result in malaria transmission, due to development of both mosquito and pathogen being highly complex [28]. However, given that the analysis was undertaken at a monthly resolution some short-term lagged correlations would be captured.

The primary strength of the analysis is the large infectious disease dataset, which is nationally representative and has information on a wide range of pathogens. We have shown how a well-known clustering algorithm (k-means) can be applied to these data to classify pathogens by their relationship with weather variables. We have utilised a number of weather parameters from the MEDMI database, which allowed for subtle differences in correlation to be illustrated. The use of two methods to detail seasonal patterns was also a strength of the analysis. The advantages of using a TBATS model is that it automatically selects Fourier terms and other aspects of the model, whilst allowing for seasonality to change over time. Wavelet analysis could be used to test for the robustness of the findings in future analysis. By sub-setting the data on the basis of seasonality detected using the difference in model fit statistics between a ‘seasonal’ and ‘non-seasonal’ model, it was less likely that the correlations with climate in the following analysis were spurious. This is akin to defining an exclusion criterion in the design of an epidemiological study to reduce the effect of bias. Having detailed the strengths and limitations of the current analysis, in the following sections we aim to explain the results in relation to previously published work under headings based on the explanations for seasonality outlined by Grassly and Fraser [3]. The data linkage was at the England and Wales level which has certain advantages (reducing noise in the data), however public health applications often require predictions at a variety of smaller scales [29]. Analysis at a local level would complement the results presented here by showing the context in which national level predictors hold.

In addition our analyses should be undertaken in different national contexts, as some pathogens shown to be non-seasonal in this context (e.g. polio, *P. vivax*) will be highly seasonal in non/under-vaccinated endemic regions.

In particular, between *Salmonella* serotypes, there was a clear hierarchy of strength of correlation with weather. The high prevalence of *Salmonella* Enteritidis (*n* = 284,761) and *Salmonella* Typhimurium (*n* = 84,204) contributed to high seasonality for these serotypes and strong associations with temperature and the auto-correlated sunshine and vapour pressure. The examination of Salmonella data showed some of the limitations that can constrain the comparison of weather and infectious disease data. While most *Salmonella* serotypes were seasonal, this could not be demonstrated for most of these until they were combined together with similar serotypes showing some evidence of more cases in summer months. The serotypes that showed no evidence of seasonality may be associated with contamination from reptiles kept as pets [30]. Such exposure is thought to be relatively less seasonal in its occurrence compared to foodborne salmonellosis. Typhoid and paratyphoid infections in England and Wales are usually associated with travel abroad, particularly to the Indian subcontinent, and this is in the late spring and early autumn [31].

### Strengths and weaknesses in relation to other studies

Temperature was most often used to explain any relationship between climate and pathogens previously [1, 32]. However, there must be careful consideration of the measure of temperature used as shown in our analysis of *Influenza A* and *B*. *Influenza A* was most strongly correlated with extreme weather events (i.e. snow lying days), which may indicate specific circumstances around these events that are important for transmission of the pathogen (i.e. temperature of below 2 °C with moisture in the air). We also found that other temperature-related variables showed consistent associations with various pathogens. Vapour pressure has been used previously in a study investigating the effect of meteorological variables on the risk of Legionnaires’ disease in Switzerland [33]. Vapour pressure may have such strong associations with several infectious diseases such as influenza [34], because it represents a set of meteorological parameters, i.e. warm, humid and wet conditions. Similar inferences were made in a study of RSV activity in the Netherlands, which found that humidity and temperature combined explained more variability than these parameters individually [35]. This may be due to the dual impact of increased contact from lower temperature and increased immunosusceptibility associated with by higher relative humidity [36]. The approach here was probably not optimal for linking waterborne diseases to rainfall because of the local linkage needed, as there are significant variations by geographic region.

### Weather and vector abundance

Weather can influence pathogen prevalence indirectly through exerting pressure on vector abundance. We found both dengue and *Plasmodium falciparum* had a seasonal pattern (although for dengue it was so weak that it was excluded at stage 1) and for the latter weak correlation with max temperature. This can be explained by rising temperatures increasing mosquito distributions and causing seasonal peaks in dengue virus and *Plasmodium falciparum* (i.e. the parasite responsible for cases of malaria) [27, 32], in the countries where the infection was likely acquired. Other native vector-borne diseases were shown to be associated with weather in the current analysis. For example, *Borrelia burgdoferi*, which infects ticks and causes Lyme disease, had a strong correlation with sunshine. *Borrelia burgdoferi* infected tick distribution was previously shown to correlate with season and rainfall in Scotland [37].

### Weather and pathogen survival

There is evidence to suggest that weather is a driver of faecal-oral infectious diseases, through the increased survival of pathogens in the environment [3]. In addition to *Rotavirus*, which have enhanced survival at low temperature, the current analysis has identified that *Aeromonas* (*A.sp, A. hydrophilia, A. sobria*), *Bacillus (B. cereus, B.* sp), *Coxsackie B*, *Cryptosporidium* sp., *Giardia lamblia*, *Listeria monocytogenes* and *Shigella sonnei* may flourish under higher temperatures. Respiratory infections transmitted by aerosols are similarly influenced by changes in weather. The high correlations between *Astrovirus*, *HMPV*, *Mycoplasma pneumoniae*, *Moraxella catarrhalis*, *Neisseria meningitidis* and *RSV*, and weather may be due to low temperatures causing increased survival and transmission or it could be lower levels of UV in the darker winter months. Further work is needed to determine if specific weather thresholds control seasonality.

### Weather and host behaviour

Weather may indirectly affect pathogen prevalence through host behaviour. Salmonella is highest in summer months which may in part be due to changes in food handling by humans during those months [11]. *Pasturella multocida*, which is caused by scratches or bites from domestic animals, was shown to be highest in July in the current analysis. Injuries caused by a cat or dog were shown to peak in summer in Bologna, Italy [38], which may be due to more time spent outdoors. As mentioned vector abundance will create higher incidence for certain infectious diseases such as malaria, dengue fever and cholera, which are then found to be higher in other countries due to travel behaviour. For example, UK travellers returning from countries with poor sanitation, typically India and Pakistan, in summer months, have an increased risk of cholera due to the seasonal effects on the pathogen growth conditions in these other countries [39].

### Weather and host immune susceptibility

Several infectious diseases are more prevalent in immune-compromised individuals. Previously it was found that patients (most of whom have medication, fluid or blood transferred using a central line catheter) were at increased risk of bloodstream infections caused by *Acinetobacter spp*., *Escherichia coli*, *Enterobacter cloacae*, *Klebsiella spp*., and *Pseudomonas aeruginosa* during summer [40]. We found associations between higher ambient temperature and *Enterobactor* (*E.* sp*., E. clocae, other named*, *E. agglomerans* (*Pantoea agglomerans)*, *Stenotrophomonas maltophilia*, *Acinetobacter baumannii*, *Psuedomonas putida* and *Pleisiomonas shigelliodes*. Mechanisms for seasonality in nosocomial infections need to be examined further to highlight whether meteorological factors are responsible for the primary infection, complications, or both [40].

## Conclusion

In this large database of infectious diseases in England and Wales, we have provided an analysis of the seasonality of common pathogens and their correlation with meteorological data. This is extremely important given the context of future climate changes. Pathogens within the 91 identified should be investigated further using the proposed meteorological variable, following recommendations proposed by Imai and colleagues [26]. In particular, future studies should be undertaken at finer spatial and temporal aggregations, using pathogen specific confounders and investigating a variety of lag effects and non-linear associations.

## Additional files

Additional file 1: **Figure S1.** Time series plots of meteorological variables. (PNG 314 kb)

Additional file 2: **Table S1.** Weekly, monthly and quarterly breakdown of pathogen seasonality. (CSV 34 kb)

Additional file 3: **Figure S2.** Seasonal pathogen cross correlations with meteorological variables. (PNG 397 kb)

Additional file 4: **Table S2.** Mean cross correlations for weather Groups. (CSV 346 bytes)

Additional file 5: **Figure S3.** Max correlation with meteorological variable by weather cluster group. (PNG 366 kb)

## Abbreviations

ADF: Augmented Dickey–Fuller
HMPV: Human metapneumovirus
KPSS: Kwiatkowski-Phillips-Schmidt-Shin
MEDMI: Medical & Environmental Data Mash-up Infrastructure project
MSL: Mean Sea Level
OPIE: Organism Patient Illness Record
PHE: Public Health England
RSV: Respiratory Syncytial Virus
SGSS: Second Generation Surveillance System
TBATS: Exponential Smoothing State Space Model With Box-Cox Transformation, ARMA Errors, Trend And Seasonal Components
