# Supplementary figures and images for: Pathogen seasonality and links with weather in England and Wales: a big data time series analysis
Source: BMC Public Health. 2018 Aug 28;18:1067. doi: 10.1186/s12889-018-5931-6 (PMC6114700; doi:10.1186/s12889-018-5931-6)

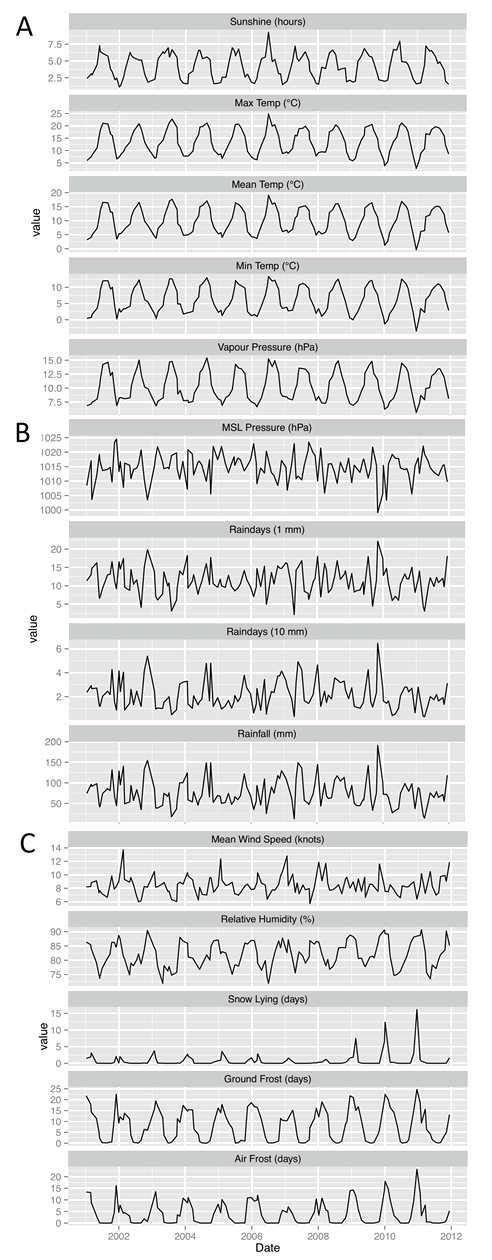

Supplement: Supplementary file 1 — Figure S1. Time series plots of meteorological variables. (PNG 314 kb) [file 12889_2018_5931_MOESM1_ESM.png]

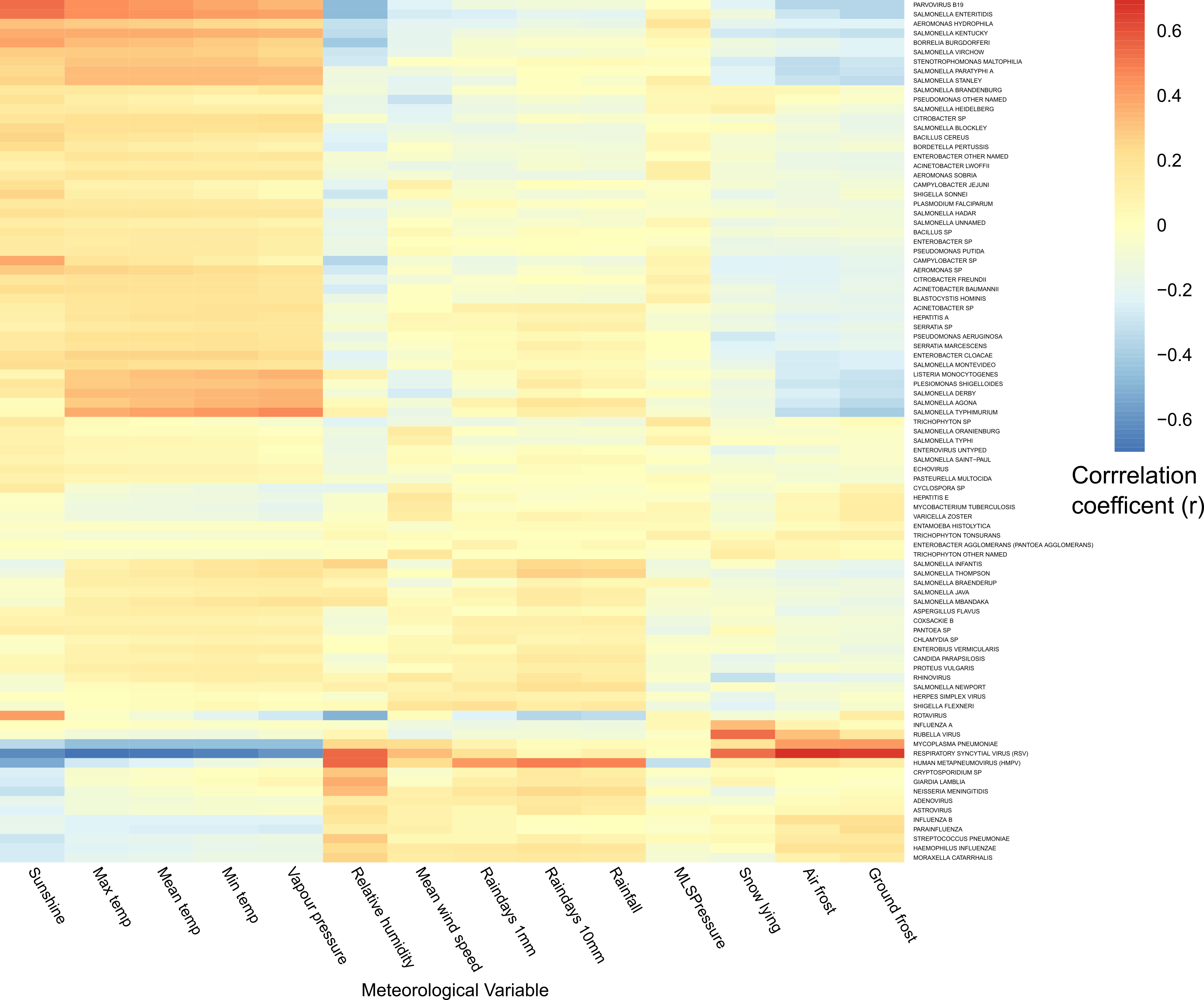

Supplement: Supplementary file 3 — Figure S2. Seasonal pathogen cross correlations with meteorological variables. (PNG 397 kb) [file 12889_2018_5931_MOESM3_ESM.png]

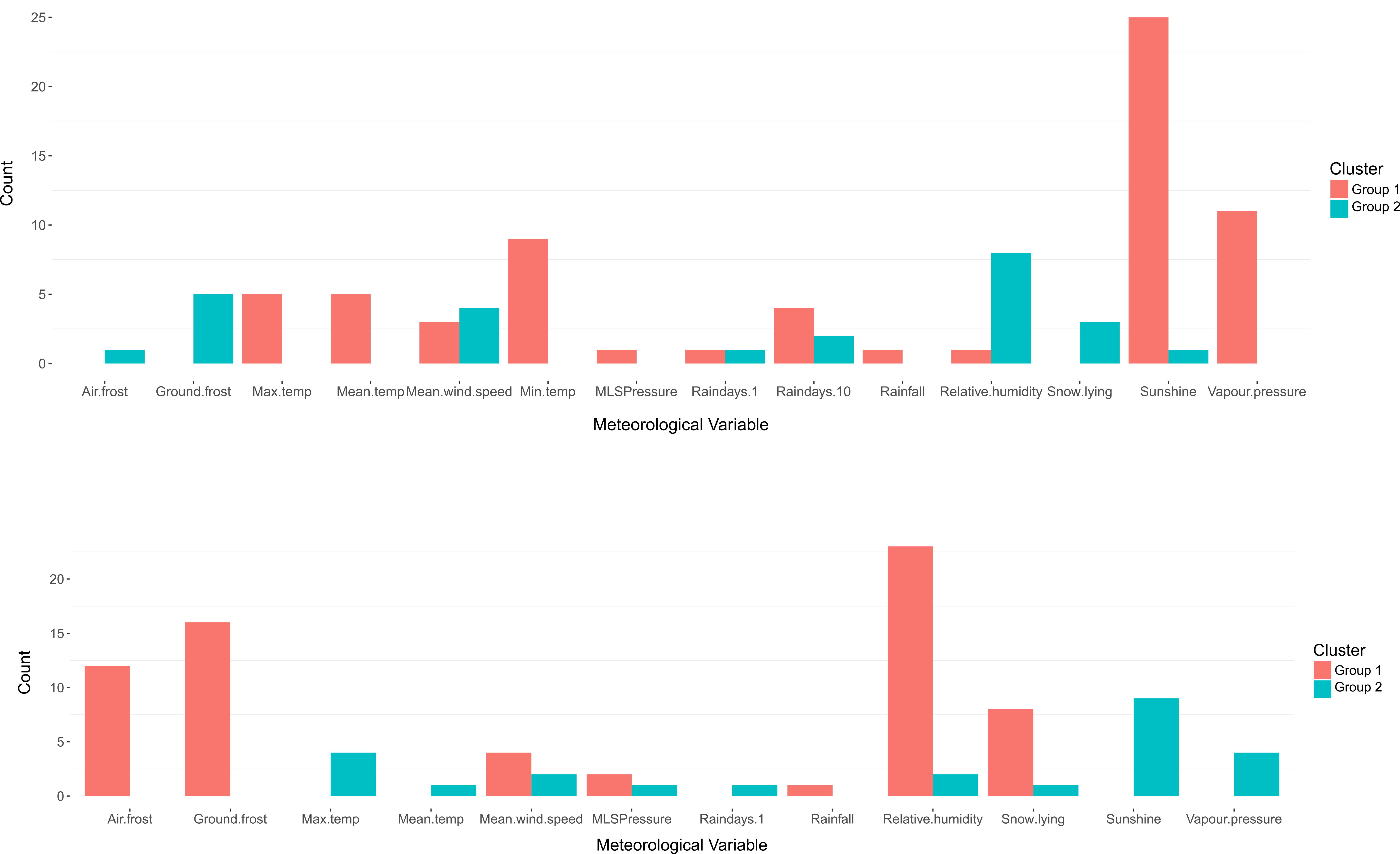

Supplement: Supplementary file 5 — Figure S3. Max correlation with meteorological variable by weather cluster group. (PNG 366 kb) [file 12889_2018_5931_MOESM5_ESM.png]
